# Assessing the utility value of Hucul horses using classification models, based on artificial neural networks

**DOI:** 10.1371/journal.pone.0271340

**Published:** 2022-07-26

**Authors:** Jadwiga Topczewska, Jacek Bartman, Tadeusz Kwater

**Affiliations:** 1 College of Natural Sciences, University of Rzeszów, Rzeszow, Poland; 2 Institute of Technical Engineering, State University of Technology and Economics in Jarosław, Jarosław, Poland; Fuzhou University, CHINA

## Abstract

The aim of this study was to evaluate factors influencing the performance of Hucul horses and to develop a prediction model, based on artificial neural (AI) networks for predict horses’ classification, relying on their performance value assessment during the annual Hucul championships. The Feedforward multilayer artificial neural networks, learned using supervised methods and implemented in Matlab programming environment were applied. Artificial neural networks with one and two hidden layers with different numbers of neurons equipped with a tangensoidal transition function, learned using the Levenberg-Marqiuardt method, were applied for the analysis. Although results showed that 7-year-old horses had the highest number of wins, the 11-year-old horses were observed to have had the best results when accessed relative to the total number of horses for a given year. Although horses from the Hroby line had the most starts in 2009–2019, those of the Goral line had the most wins. While predicting the horses’ efficiency for the first 6 positions during the utility championship, the neural network consisting of 12 neurons in hidden layer performed the best, obtaining 69,65% efficiency. The highest horse efficiency classification was obtained for the four-layered network with 12 and 8 neurons in the hidden layers. An 81.3% efficiency was obtained while evaluating the correctness of the prediction for horses occupying positions 1 to 3. The use of AI seems to be indispensable in assessing the performance value of Hucul horses. It is necessary to determine the relation between horses’ traits and their utility value by means of trait selection methods, accompanied with expert advice. It is also advisable to conduct research using deep neural networks.

## Introduction

The Hucul horses constitute a very valuable population, whose features have, as far back as the 15th - 16th century [1] been shaped by the prevailing environmental conditions of the Eastern Carpathians. This breed was nurtured, based on local populations of primitive tarpan and Przewalski’s horses, and ennobled with stallions of many noble breeds. This alpine breed is distinguished by a number of traits such as hardiness, adaptability to difficult terrains, very good feed conversion, longevity and fertility. Its exploitation in difficult mountain terrain required above-average physical fitness and a very good locomotor apparatus [1, 2]. As late as in mid-20th century, the Hucul horses were scarce and its preservation was simply due to the involvement of the breed’s enthusiasts. In 1979 a resolution regarding the necessity to protect the Hucul breed as a national heritage, nature conservation and consolidation of the valuable genotypes paved way to undertake research on the breed and to source finances from national funds in support of the breeders [3]. They have, since Poland’s accession to the European Union in 2004, been protected under the Livestock Genetic Resources Conservation Program—agri-environmental package [4]. These activities contributed to an increase in the population size, but it did not exceed a level that can be considered safe from possible genetic erosion process [5]. The need to intensify biodiversity conservation efforts has been emphasized in many studies [3, 6–8]. Conservation and use strategies require interdisciplinary research, and the use of innovative tools to compliment traditional ones should contribute to the conservation and sustainable use of Hucul horses [7, 9, 10].

Artificial intelligence methods have been applied to the study of many phenomena and problems, both in agricultural and biological sciences. Scientific and technological progress using artificial intelligence in agriculture, as reported by Geli et al. [11] and Linaza et al. [12], relies on overcoming interdisciplinary barriers as well as its accessibility to all agricultural sectors equally. Innovative solutions for short-term and long-term management are needed, especially for natural resources and biodiversity conservation.

Systems based on artificial intelligence have continued to evolve as research is undertaken in many fields of agriculture, including agronomy and animal husbandry. Horse studies have included, among others, estimating the need for surgery and the probability of survival in case of colic occurrence [13], biomechanical studies for gait phenotyping for breeding and genetic studies [14], predicting the performance value of Hucul horses [15], pain identification based on horse’s facial activity [16] and behavioral changes [17], as well as the occurrence of lameness based on movement trajectory [18]. The popularity of horse racing has contributed to a number of studies involving the prediction of race results [19–22], the identification of Arabian racehorses [23], and the optimization of a decision management model for competition organization [24].

The selection and choice of best individuals constitute a key criterion for the preservation of the Hucul breed. The program for evaluating the utility and breeding value of the breed takes into account the specific conditions in which it was reared as it is one of the elements in the verification of its characteristic traits. A key issue is, in the case of small animal populations threatened with extinction, the assessment of their breeding and performance value, especially at the stage of selection and chioce of individuals for mating. Similarly, it should be emphasized that only a minor percentage of the Hucul horse population is usually subjected to such evaluation within the framework of the breed championships [15]. Thus, the development of a tool that allows the indication of characteristic traits and factors important for the evaluation of the breeding and performance value of Hucul horses to resolve this problem is deemed necessary. In consequence, the aim of the study is:

- to evaluate factors influencing the performance of Hucul horses;
- to develop a predictive model based on artificial neural networks to predict the classification of horses, that are subjected to performance value assessment value, during the annual Hucul championships;
- to elaborate on a method (tool) that enable the assessment of the performance and breeding value of the Hucul horses, based on the description of their characteristic traits. The measure of the subjective value of the horses refers to the results obtained by them at the championships. Using the collected data, a feedforward multilayer artificial neural networks was constructed, which on the basis of selected horse characteristics and the obtained results classifies horses into three value groups.

## Material and methods

The analysis comprised results of Hucul horses obtained during performance championships for the breed from 2009 to 2019. Horses at the age of 4 are legible for the performance championships, in keeping with the Hucul horses’ genetic resources protection and breeding programs [4]. The performance championship consists of three elements, including general assessment, Hucul path and the endurance-condition test. The exterior of the horse is judged anonymously by at least three independent judges. Horses are presented hand-led on a board in a standing position and at a walk and trot in the show ring—a triangle 30 x 40 x 30 meters in size. The Hucul path is performed over a distance not exceeding 5000m. Besides the faultless scaling of the obstacles, there are sections on the trail route that should be completed using the recommended gait. The panel of judges specifies the pace at which the Hucul path, that includes up to 30 natural or artificial obstacles should be completed. The rally, also referred to as condition trial, is a test of the horse’s endurance and preparation. It is held over a distance of 15–20 km for a specified duration at the speed of 11–12 km/h. The guidelines specify, in detail, the rules of completing the route. After arriving at the finish line, the horse undergoes one veterinary examination, which should be carried out within 20 minutes of crossing the finish line. The horse’s pulse rate during the examination should not exceed 64 beats/min.

A horse’s exterior can be evaluated for a maximum of 50 points, while the number of points for successfully completing the Hucul path and endurance-condition test is 80 points each. Conversion factors are adopted for each element, 0,8, 1,25 and 0,75 respectively in the rules for the performance championships. The maximum number of points that a pair of participating horse can obtain during the performance championship is 200 points. The right to participate in the National Hucul Utility Championship is determined by a horse’s ranking, resulting from its participation in the qualifying Hucul path. The number of qualifying Hucul path, their detailed rules and regulations are decided at the meeting of the Stud Book Commission and the Polish Horse Breeders Association, usually in March, of the championships year.

The data exploited in the study and the results obtained by Hucul horses during the breed’s performance championship are published after the competition and are available on the competition organizer’s website. Additionally, detailed information about each individual horse is made available in the competition catalog, which also contains the rules and regulations guiding the competition. The results of 184 horses were considered in the analysis. The competition regulations for horses in utility championships do not limit multiple participation. The database consisted of 374 results (S1 Table) since some horses competed more than once in the Hucul utility championship during the period 2009–2019.

### Research methodology

The data obtained from Hucul horses’ starts in championships included data from 11 years, with each starting horse being characterized as follows:

- 11 descriptive traits that are objective in nature with no evaluation (name, sex, year of birth, age, coat, father, lineage, mother, family, breeding, owner);
- 5 numerical features resulting from expert evaluations during the competition (type, conformation, walk, trot, overall impression);
- finishing position during the competition.
- During the first stage of the research, determining traits were selected, which were applied in the prediction and classification process. It was decided that at this stage only descriptive traits with no evaluations will be used. Establishing a correlation between horse traits and horse performance is not easy, because most traits are not numerical, but descriptive, such as coat colour, pedigree, family and breeding. Hence, in order to determine the correlation, numerical values had to be assigned to individual traits. Assigning numerical values to features should not be arbitrary, since it can blur existing correlations. This calls for the application of heuristic knowledge in such instances. The simple analysis undertaken resulted in the rejection of the horse’s name as a trait that determines its performance, its year of birth as it relates to the horse’s age, father and mother lineage due to its negligible repeatability from out of the 11 descriptive traits earlier outlined. The remaining traits were tested to determine if they have any effects on performance.

In order to evaluate whether the genealogical affiliation of horses (line and family) has any influence on their results, support indicators were introduced for the purpose of the research:

- the coefficient of efficiency of starts by horses from a given pedigree in single competitions:

$$SR=\frac{1}{n}\frac{1}{m}$$ (1)

where: n—the number of horses from a given pedigree starting in the competition in a given year;

m–horse’s position in the competition.

The coefficient value attains its maximum 1, when a horse from a pedigree starts and finishes in first position, the more representatives of a pedigree that start and the lower the position they finish in, the lower the SR coefficient value;

- the line’s average starting efficiency coefficient that enables the assessment of the line’s efficiency within an assumed time interval to be carried out, based on:

$$ASR=\sum_{i=1}^{k}\frac{1}{n}\frac{1}{m}\frac{1}{k}$$ (2)

where: n, m—as explained earlier, k—number of competitions.

The ASR coefficient is a weighted sum of SR coefficients from individual competitions, in which horses from a given lineage took part.

The next stage of the research involves building an artificial neural network (SSN), used to predict the positions taken by horses in competitions. It makes direct use of data describing the horses.

The last stage is the classification of horses assigned to any of three groups: strong, medium, weak, based on their characteristic features such as age, genealogical affiliation (line and family), sex, coat colour, breeding region and ownership (private or public). As a classifier, a feedforward multilayer artificial neural networks was used, learned using the supervised method, whose input vector were the aforementioned characteristic features, and whose output vector was defined based on the historical results of competitions in which the horses had participated.

Due to the lack of unanimity of the terms "strong", "medium", "weak", it was decided that the description applicable in fuzzy sets be used to define the output vector, defining each group with a pair of numbers {(*x*, *μ*_*A*_(*x*)}, where *x* denotes the value of the element (horse’s position in the competition), whilst *μ*_*A*_(*x*)∈p0,1] defines its coefficient of membership of the set *A*.


$$Strong(x)=\left\{ \begin{array}{l} 1\;dla\;x\leq 3\\ 1-\frac{x-3}{10}dla\;x>3\;i\;x\leq 12 \end{array}\right.$$ (3A)

$$Mean(x)=\left\{ \begin{array}{l} 1-\frac{13-x}{10}\;dla\;x>3\;i\;x\leq 12\\ 1\;dla\;x>12\;i\;x\leq 23\\ 1-\frac{x-23}{10}dla\;x>23\;i\;x\leq 32 \end{array}\right.$$ (3B)

$$Weak(x)=\left\{ \begin{array}{l} 1-\frac{33-x}{10}dla\;x>23\;i\;x\leq 32\\ 1\;dla\;x>32 \end{array}\right.$$ (3C)

**Fig 1:**
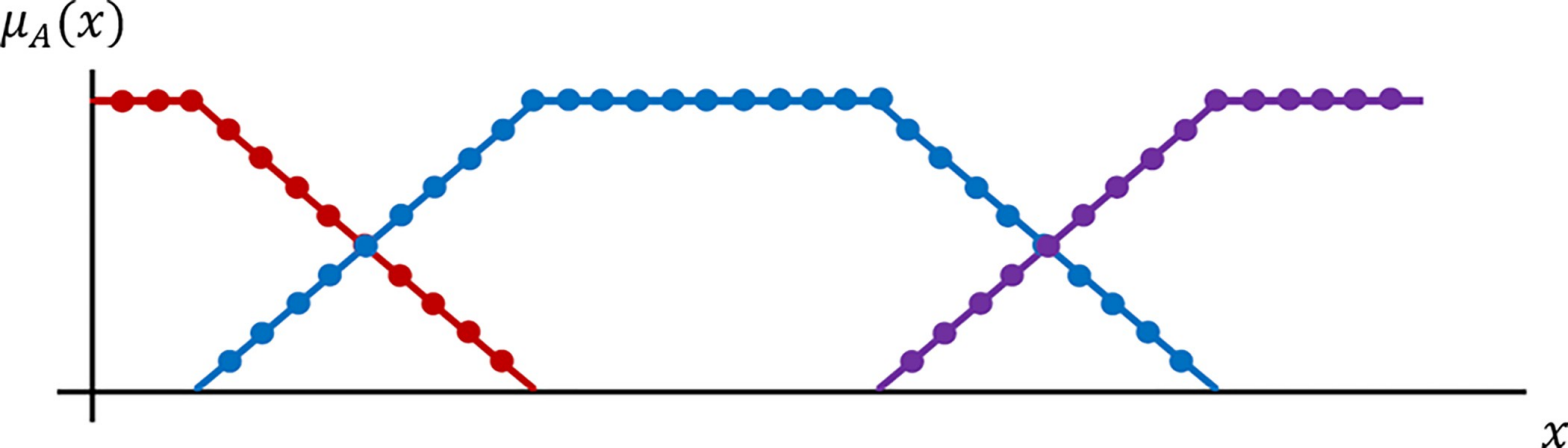
Components’ membership of groups „strong”, „medium”, „weak”.

Three set were thus defined (Fig 1):

### Configuration of the artificial neural network

The specificity of the difficulties associated with the study, characterized by a high degree of vagueness, indicates that the most appropriate tool for their solution will be through the use of Artificial Intelligence methods, specifically Artificial Neural Networks (ANN). The choice of ANNs is dictated by their properties, which in the case of the conducted research may include such relevant ones as:

- suitability for learning, based on examples,
- suitability for the generalization of phenomena (classification),
- suitability for the interpretation of relations and phenomena containing incomplete and inconsistent information.

Multilayer unidirectional networks learned by supervised methods were selected from amongst many existing types of artificial neural networks. This type of network performs well in prediction and classification processes [18, 25]. Supervised learning was chosen because of the availability of historical data concerning horses’ participation in competitions, and the awareness that supervised learning is more efficient than unsupervised learning [26].

The choice of horse traits used for predicting and classifying horse was preceded by preliminary tests to determine whether a given trait impacts on the results obtainable by horses.

The proposed solutions, neural prediction and neural classification systems make use of multilayer artificial neural networks implemented in the Matlab programming environment (Version R2020b).

**Fig 2:**
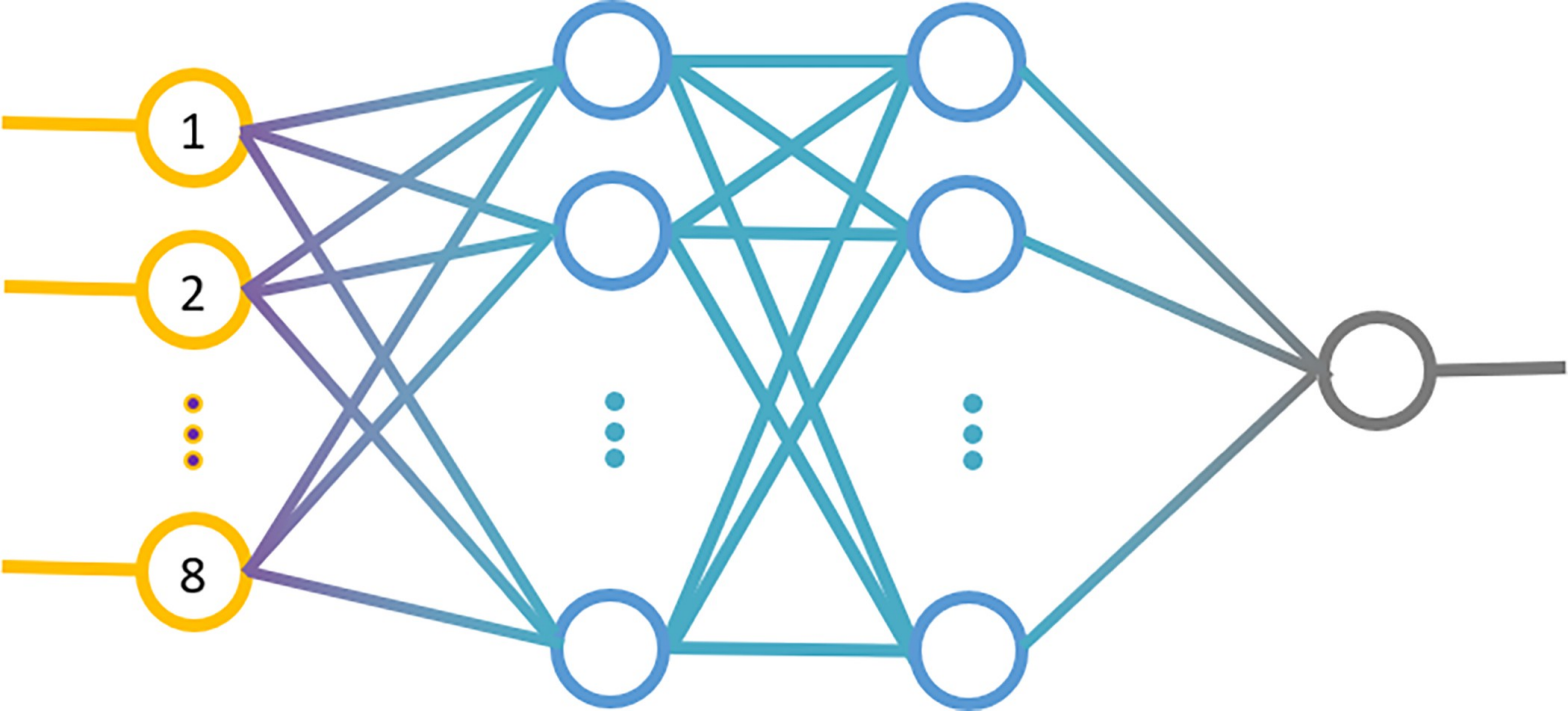
An illustration of the network used to predict the horses’ position in the championship.

For the predictive system, the research was carried out for networks with one hidden layer consisting of 8, 10 or 12 neurons equipped with a tangensoidal transition function and for networks with two hidden layers consisting of 7 and 5 neurons, 8 and 4 neurons and 9 and 3 neurons with identical transition function (S1 File, Fig 2).

The learning process (update weight and bias states, according to Levenberg-Marquardt optimization) was performed, using the Levenberg—Marquardt (LM) method, being the standard recommended for Matlab environment (where MATLAB Neural Network Toolbox is used to learn the parameters in the network). In order to evaluate the predictive capabilities of the network, a test was performed on a sample of data that was not used for learning. Hence, the dataset was divided into two parts: learning (2/3 of the set) and testing (1/3 of the set). Due to the set’s small size, a cross-validation was first applied, followed by the averaging of results in order to obtain reliable and stable results. With respect to the classification system (used to assign membership to any of the three groups), however, the network with one hidden layer was applied in the research.

## Results

### Selecting horses’ representative traits–testing dependencies between a horse’s characteristics and results obtained

**Fig 3:**
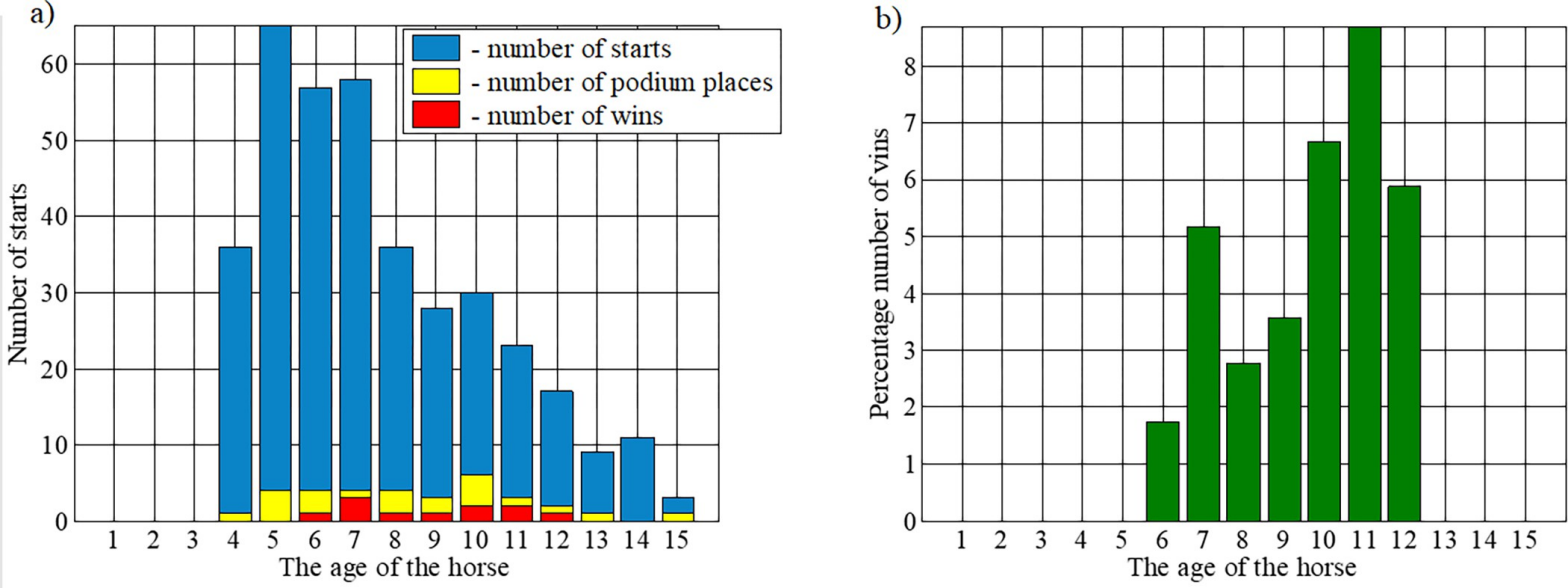
Number of horse victories according to their age. a) absolute values, b) percentage values.

The data analysis indicated that the results obtained depend on the age of the horse. Despite being impossible to indicate precisely the horse’s optimal age, horses aged 5 or 6 years can be considered to have performed well, taking into consideration the top 6 positions. On the other hand, if only victories were considered, then utility championships were won by horses aged 6 to 12 years (Fig 3A, Table 1).

**Table 1:** Summary of position occupied by horses, according to age.

Age	Place						Average place	Number of horses
	1	2	3	4	5	6		
4	-	1			2	1	4	19,9
5	-	2	2	1	2	3	**10**	18,2
6	1	1	2	3	1	2	**9**	17,7
7	**3**	-	1	2	2	-	8	19,0
8	1	2	1	1	1	-	7	17,2
9	1	1	1	1	-	-	3	19,2
10	**2**	2	2	-	-	2	7	**12,9**
11	**2**	-	1	2	-	1	7	**14,9**
12	1	1	-	-	2	1	5	18,6
13	-	-	1	-	1	-	2	16,1
14	-		-	1	-	1	2	16,5
15	-	1	-	-	-	-	1	-

The majority of victories were by 7 year-old horses. However, if the number of wins were compared to the total number of horses at a given age, 11 year-old horses were the best (Fig 3B, Table 1). When, on the other hand, the average results for a given age group were analyzed, it was found that the best results were achieved by 10 and 11 year-old horses (Table 1).

**Fig 4:**
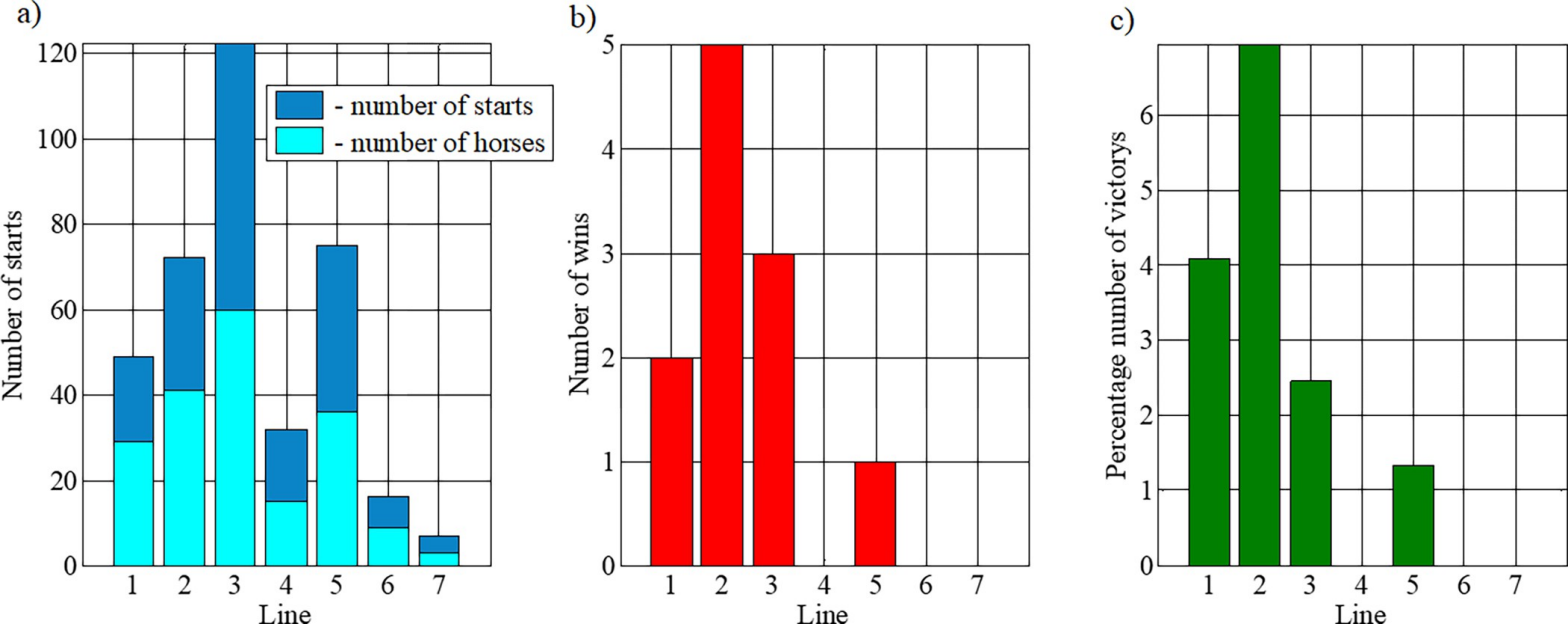
Participation in competitions by line: a) total number of horses from a line participating in competitions, b) number of victories of horses from the line, c) percentage number of victories (number of victories of the line related to the number of competitions). The line were marked with numbers: 1—Gurgul, 2—Goral, 3—Hroby, 4—Oušor, 5—Pietrosu, 6—Polan, 7—Prislop.

In evaluating the influence of the pedigree on the results, it is worth noticing that the highest number of starts was by horses belonging to the Hroby line (Fig 4A), whilst the highest number of victories, both numerically (Fig 4B) and in percentage (Fig 4C) was recorded for the Goral line. If only the victories were considered, the line can be ranked successively, starting with the best: Goral, Gurgul, Hroby, and Pietrosu.

**Fig 5:**
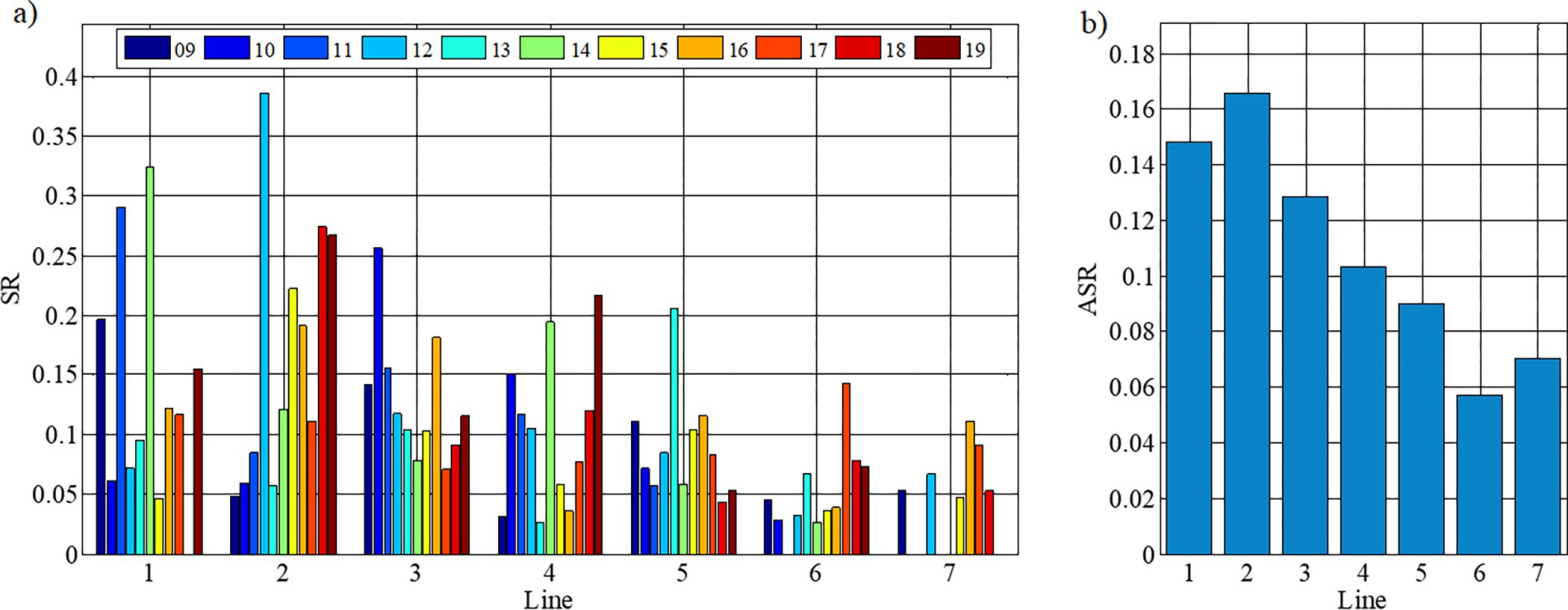
Values of pedigree effectiveness factor in a given year (SR) and the average value of the pedigree effectiveness factor from all starts (ASR). The line were marked with numbers: 1—Gurgul, 2—Goral, 3—Hroby, 4—Oušor, 5—Pietrosu, 6—Polan, 7 –Prislop.

It was observed that for a specific stallion line the SR coefficient varies from competition to competition, with the most stable results being obtained by horses from the Hroby, Polan and Prislop lines (Fig 5A). The highest average starting performance (ASR) (Fig 5B) was achieved by the Goral line followed by Gurgul, Hroby, Oušor, Pietrosu, Prislop and Polan successively (Fig 5).

**Fig 6:**
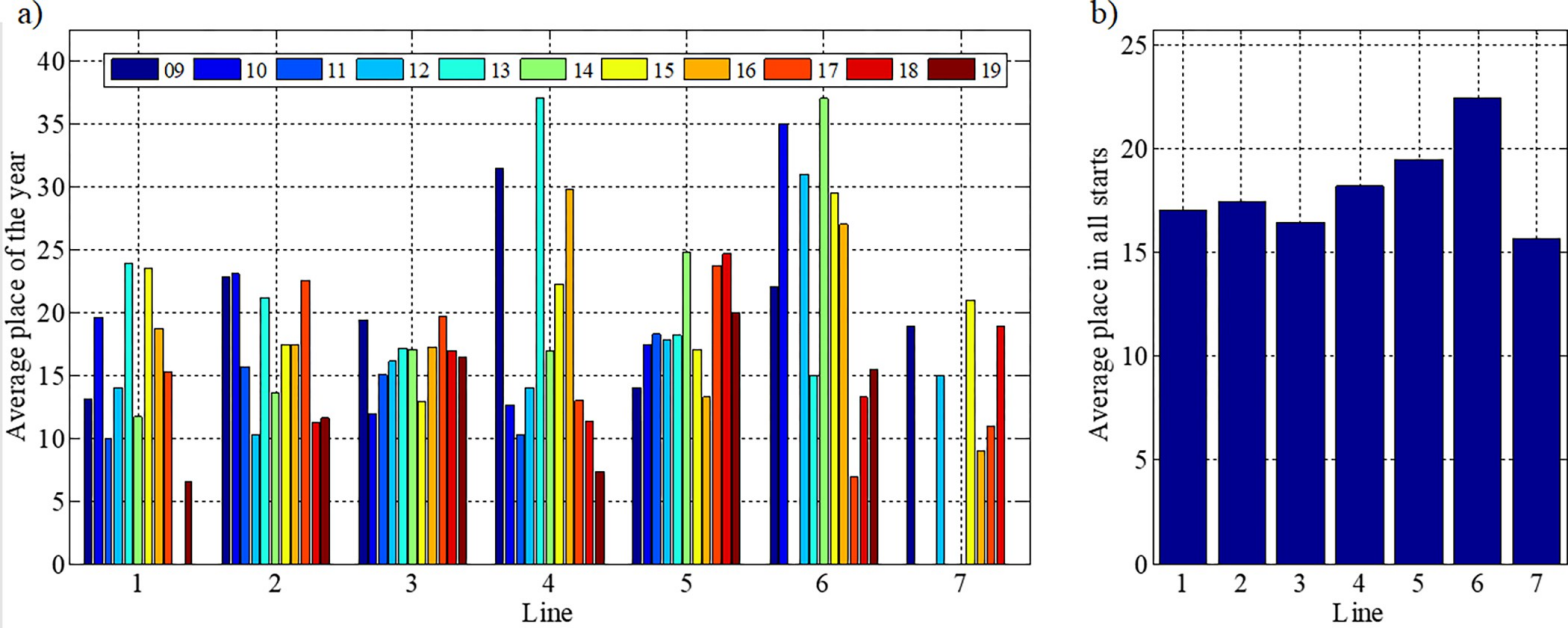
Positions of horses competing in Hucul championships in 2009–2019. The line were marked with numbers: 1—Gurgul, 2—Goral, 3—Hroby, 4—Oušor, 5—Pietrosu, 6—Polan, 7 –Prislop.

Fig 6 shows the average position of horses during starts, as representatives of the line in a given year. The most stable results were achieved by horses from the Hroby line. However, in the case of the horses’ average position from the starts, according to their pedigrees from all competitions in 2009–2019, it was found that the best were successively by individuals from Prislop, Hroby, Goral, Gurgul, Oušor, Pietrosu and Polan lines.

The results obtained indicate that belonging to a pedigree has an influence on the horses’ results. However, it is not possible to indicate the lineage that is the absolute best, since differences in indications obtained depend on the criterion adopted.

By analogy, tests were conducted for the other traits: sex, coat, family, breeder and owner. The results obtained indicate that each of these traits may have an influence on the results obtained. Hence, their inclusion in future studies into learning data is encouraged.

### Prediction of horse classification—testing a prediction model based on artificial neural networks

The performance of the network predicting the horse’s position in a competition is presented in percentage correctness of indication. The effectiveness of the network’s performance, understood as the indication of the correct position of the horse during the performance championships of Hucul horses is presented in Table 2. The best results were generated by the network composed of two hidden layers with 9 and 3 neurons and it was 24.66% error-free results. On the other hand, the network with 8 and 4 neurons also proved to be the best in predicting the position of the horse in the competition with an accuracy to the third decimal place, resulting in an efficiency of 53.39%. The best position prediction results (69.65%) with an accuracy of 6 decimal places were generated by the network composed of one 12 neuron hidden layer.

**Table 2:** Results of predicting horses’ positions in competitions.

Error	Number of neurons in hidden layers					
	8	10	12	7 & 5	8 & 4	9 & 3
0	15,18	17,62	17,07	22,22	**24,12**	24,66
1	8,13	11,65	13,82	12,74	13,28	12,20
2	4,61	10,57	10,03	7,86	8,13	6,78
3	10,57	8,67	7,59	8,40	7,86	6,50
Σ(0–3)	38,48	48,51	48,51	51,22	**53,39**	50,14
4	8,94	6,23	9,49	4,61	4,61	4,61
5	7,59	4,61	6,50	5,15	4,88	4,61
6	5,69	3,25	5,15	4,34	3,52	4,61
Σ(0–6)	60,70	62,60	**69,65**	65,31	66,40	63,96

The neural network was tested using the index:

$$Accuracy=\frac{correct\;clasifications}{all\;clasifications}$$ (4)

### Assigning horses to strong, medium, weak groups—testing a classification model based on artificial neural networks

The output vector obtained consists of three quantities defining the value of the coefficient of belonging to each of the extracted groups. The group for which the maximum value was obtained in the response was taken as the classifier indication. The highest classification efficiency for the whole data set was obtained for the four-layer network with 12 and 8 neurons in the hidden layer and it was 81.8%. The evaluation of the correctness of the classification of horses occupying positions 1 to 3 in the "strong" group, showed efficiency of 81.3% in the four-layer network composed of 14 and 6 neurons in the hidden layer (Table 3).

**Table 3:** Results of horses’ classification into groups "strong", "medium", "weak".

Number of neurons in hidden layers	Efficiency of identification	
	whole data set	1 to 3 in the "strong" group
8	33,3	69,4
12	57,6	75,3
16	57,4	80,0
10–4	57,6	76,4
12–6	72,7	78,3
12–8	**81,8**	80,2
14–6	69,7	**81,3**
14–7	69,7	80,0

## Discussion

The Hucul horses, reared in Eastern Carpathians, are an important European cultural asset. The Polish breed of the horse constitute the largest population of this breed. At the same time Poland is the leader in terms of involvement in pedigree and sire selection, systematic verification of performance value among the countries associated in the European organization of Hucul International Federation (HIF). The Polish Horse Breeders Association maintains, by permission of the HIF, the Hucul Breeders’ Book of Origin in accordance with the European Commission Decision 92/353/EEC of 11 June 1992, which lays down criteria for the recognition or legalization of breeding organizations and associations. Its goals include, among others, the preservation of original traits, as well as insisting that all breeding activities conform with the European scale (Book of Origin of the Hucul Breed, PZHK). Performance championships for Hucul horses, that have been organized in Poland for over twenty years, are key instruments for improving the breed and controlling the preservation of its typical traits.

Various approaches and methods were used in the analysis to obtain results concerning the prediction of the Hucul horses’ performance values and its determining factors. The determination of the optimal age for the assessment of the utility and breeding value is of key significance. The lack of unambiguity of indications in this respect may result from the limited data size. This is also observed while selecting the family or families obtaining the best results (Fig 6). At the same time, too little number of horses belonging to the breed are entered for participation in qualifying trails and in consequence for participation in the All-Polish performance championship [15].

The application of artificial neural networks has made the analyses of complex phenomena or problems possible. An important issue regarding the limitation of rising inbreeding coefficient and the preservation of genetic variability, especially with respect to small populations, is assessing their utility and breeding values, as well as the proper selection of individuals for mating. Genetic erosion remains a major threat to the Hucul breed. Another issue could be the preservation of the appropriate genetic variability by maintaining an adequate representation of all the male and female families, among other things. The use of artificial neural networks allows us to take advantage of their ability to interpret relationships and phenomena containing incomplete and inconsistent information, that researchers have to contend with working on small populations.

The accuracy outcome of 24.12 for the position occupied obtained in the analysis performed using artificial neural networks may not seem satisfactory, but when considered in respect of predicting positions for the first 3 places, the outcome indicates an accuracy of over 50%. By increasing the prediction error for position accuracy indication up to 6 places, it was possible to achieve a 70% accuracy. Kil et al. [17], using deep learning to analyze changes in horse behaviours representing pain responses, obtained a result of over 80%. In contrast, using machine learning to classify horses’ gaits, Serra Bragança et al. [14] obtained 97% accuracy. Similarly, very high accuracy was obtained by van Steenkiste et al. [27] for a network that made use of a parallel convolutional neural network architecture to classify ECG recordings for the diagnosis of cardiac arrhythmias in horses.

Promising results were obtained in our research models using fuzzy sets and neural networks, in which 81.8% of horses occupying positions 1–3 in the championship were classified into the "strong" group by a classifier that uses objective features. Since the indicated factors, deemed important in the classification of Hucul horses can be generalized for an entire population, they offer possibilities of use both for the needs of selection and choice of individuals for matchmating.

## Conclusion

Summing up, the research undertaken indicates that the use of statistical methods and artificial intelligence methods do seem indispensable in the assessment of the Hucul horses’ utility value. It is necessary to determine the relationship between horses’ traits and their utility value using trait selection methods (Hellwig, Relief, etc.) with expert knowledge. It is also advisable to conduct research using deep networks. However, the realization of these intentions requires, first and foremost, the enlargement of the data set.

## Supporting information

S1 Table The database. (PDF)

S1 File (PDF)
