# Supplementary material for: Assessing the utility value of Hucul horses using classification models, based on artificial neural networks
Source: PLoS One. 2022 Jul 26;17(7):e0271340. doi: 10.1371/journal.pone.0271340 (PMC9321442; doi:10.1371/journal.pone.0271340)
Supplement: S1 Table — (PDF) [file pone.0271340.s001.pdf]

horses	sex	birth	year of chem|	line	family	type	conformati	walk	trot	overall impres
afisz	o	2000	2009	prislop	agatka	7,2	7	7,6	7,2	8
akacja-w	k	2005	2009	cukor-gurgul5	agatka	9	7,6	7,2	8,2	8,6
antaba	k	2004	2009	cukor-gurgul5	agatka	8,8	8,2	7,8	7,8	9
bercik	o	2004	2009	goral	bajkałka	9,2	7	6,6	7,6	8
gejzer	o	2002	2009	polan	gurgul V-23	8,4	7	7	7,6	8,2
grażda	k	1995	2009	goral	gurgul V-23	9,8	8,2	9	8,2	9,8
hewia	k	2001	2009	hroby V	polanka	7,8	7	7	7,6	8,8
hydra	k	2004	2009	cukor-gurgul5	polanka	7,4	7,8	7	8	8,4
jamajka	k	2003	2009	pietrosu VI	jagoda	8,8	8	7,8	7,6	8,2
juma	k	2004	2009	hroby V	jagoda	9,2	8	7	8	9
leda-o	k	2004	2009	goral	laliszka	9,6	7,8	8	8	9
liana	k	1999	2009	pietrosu VI	laliszka	7,4	7,2	8	8,8	8,2
lotnik	o	1998	2009	ousor VIII	laliszka	9	8	8,6	9	9,4
nagan	o	2004	2009	pietrosu VI	nakoneczna	8	7	7,4	7,8	8,8
narcyz	w	1998	2009	cukor gurgul-5	wyderka	8	8	7,2	6,8	8,4
nasir	o	2003	2009	cukor-gurgul5	nakoneczna	9,4	8,4	7,8	8,2	9,4
oliza	k	2001	2009	cukor gurgul-5	nakoneczna	8,4	7	7,2	7,8	8,8
opończa	k	2003	2009	pietrosu VI	wołga	8,8	8	8,2	8	8,8
otto	o	2000	2009	goral	wołga	8,6	7,4	7,2	7,2	8
pablo	w	2002	2009	hroby V	polanka	6	7	7	8	7,6
pandora-tabun	k	2005	2009	pietrosu VI	polanka	7	7,2	7,8	7,8	8,4
patafian	w	2005	2009	pietrosu VI	polanka	6,4	7,2	6,8	6,8	7,4
powiew	o	2000	2009	hroby V	polanka	8,6	7,4	9	7,4	9
pozerka	k	2004	2009	goral	polanka	7,4	7,2	6,4	7,8	7,8
pralinka	k	1999	2009	cukor gurgul-5	polanka	8,4	8	9	8,2	9,6
sobótka	k	2003	2009	cukor-gurgul5	sroczka	10	8,2	9	8,2	10
suprima	k	2002	2009	pietrosu VI	sroczka	7,2	7,2	8	7,8	8,6
suzi	k	2005	2009	pietrosu VI	sroczka	8,2	7,8	8	8,2	9
sybira	k	2000	2009	goral	sroczka	8,8	8	7,8	8	9,2
szlak	w	2004	2009	pietrosu VI	sroczka	8	8	9	8	9,8
wanta II	k	1996	2009	cukor-gurgul5	wołga	8	8	8,2	7,8	8,6
wega	k	2002	2009	cukor-gurgul5	sroczka	9	7,6	8,6	7,8	8,8
wicher	o	2004	2009	ousor VIII	wyderka	9	8	8	7,4	8,2

wisia	k	2002	2009	hroby V	wyderka	7,6	7,2	7,2	7,8	7,8
akacja-w	k	2005	2010	cukor-gurgul5	agatka	8	7,6	7,8	8	8,5
aksa	k	1995	2010	cukor-gurgul5	jagoda	7,5	7,2	7,2	7,1	8,1
biela	k	2003	2010	cukor-gurgul5	cocha	7	6,6	7,8	7,8	7,9
borówka	k	2003	2010	goral	wrona	8,5	8,1	7,9	7,9	8,7
gejzer	o	2002	2010	polan	gurgul V-23	8,1	7,7	7,1	7,9	7,9
grenada-o	k	2004	2010	cukor-gurgul5	gurgul V-23	8,1	7,7	8,4	7,7	8,4
greta	k	2003	2010	hroby V	gurgul V-23	8,3	8	7,7	7,7	8,8
iskra	k	2003	2010	pietrosu VI	jagoda	8,5	7,6	7,8	7,4	8,2
jokasta	k	2005	2010	goral	jagoda	7,5	7,8	8,1	7,5	8,2
laguna-o	k	2005	2010	hroby V	laliszka	8,3	7,7	7,7	7,3	8,6
lando-o	o	2004	2010	hroby V	laliszka	8,5	7,7	8,7	7,9	8,5
liana	k	1999	2010	pietrosu VI	laliszka	7,7	7,4	8,5	8,1	8,3
lotnik	o	1998	2010	ousor VIII	laliszka	9,4	8,2	8,8	8,7	9,1
nagan	o	2004	2010	pietrosu VI	nakoneczna	7,7	7,3	7,6	7,7	8
nasir	o	2003	2010	cukor-gurgul5	nakoneczna	9,2	8,5	8,7	8,2	9,4
neron	w	2003	2010	hroby V	nakoneczna	7,6	8,1	7,6	7,4	8,4
oficer	o	2005	2010	hroby V	wołga	8,5	7,8	7,2	7,3	8,4
okowita	k	2003	2010	cukor-gurgul5	wołga	9,6	8,8	8	7,4	9,3
olgierd-o	o	2005	2010	ousor VIII	wołga	9,2	8,3	7,6	7,7	8,9
oliwka	k	2005	2010	hroby V	wołga	9,4	8	8,5	7,8	9,4
otto	o	2000	2010	goral	wołga	8,7	7,9	6,9	6,5	7,9
paługa	k	2006	2010	goral	polanka	8,3	7,7	7,8	7,7	8,7
patka	k	2006	2010	hroby V	polanka	8,5	7,8	8,1	8	8,8
powiew	o	2000	2010	hroby V	polanka	8,2	7,7	7,5	7	8,5
prowizja	k	2005	2010	goral	polanka	8,9	8	7,6	8,2	8,9
sobótka	k	2003	2010	cukor-gurgul5	sroczka	9,5	8,2	8,8	7,9	9,4
sowa-kur	k	2006	2010	cukor-gurgul5	polanka	8	7,9	7,5	7,2	8,5
suprima	k	2002	2010	pietrosu VI	sroczka	8	7,7	7,8	7,2	8,9
wanta II	k	1996	2010	cukor-gurgul5	wołga	8	8	8,3	8,5	9
wega	k	2002	2010	cukor-gurgul5	sroczka	9,3	8	8	7,3	8,5
wielkanoc	k	2003	2010	goral	wyderka	6,2	6,2	7,2	7,2	7,2
wigilia	k	2000	2010	ousor VIII	wyderka	7,9	7,8	8,4	7,8	8,5
wisia	k	2002	2010	hroby V	wyderka	8,5	8,1	8	7,8	8,6

azalia-w	k	2006	2011	ousor VIII	agatka	9	8,3	8,7	8,7	9,5
bogacz	o	2007	2011	hroby V	bajkałka	7,5	7,3	8,7	7,8	9
fala	k	2003	2011	hroby V	wrona	8,7	7,8	7,8	8	9
fifka	k	2007	2011	hroby V	wrona	8,5	8	8	7,5	9
istria	k	2007	2011	hroby V	jagoda	8	7,3	8,3	8	8,8
jagła	k	2006	2011	goral	jagoda	8,7	8,2	8,2	8,2	9,3
juma	k	2004	2011	hroby V	jagoda	8,5	7,8	7,8	7,7	9
laguna-o	k	2005	2011	hroby V	laliszka	8,2	7,5	7,5	7,7	8,8
lando-o	o	2004	2011	hroby V	laliszka	8,5	7,5	8,2	8	9,3
leda-o	k	2004	2011	goral	laliszka	8,5	8	7,5	7,2	8,3
oficer	o	2005	2011	hroby V	wołga	9	8,5	7,5	7,5	9,7
ogień	o	2007	2011	hroby V	wołga	8,3	7,5	7,2	7	7,8
okowita	k	2003	2011	cukor-gurgul5	wołga	8,5	7,7	7	7,3	8,2
oleśnica	k	2003	2011	hroby V	wołga	9	8	7,7	8,3	9
olgierd-o	o	2005	2011	ousor VIII	wołga	9,2	8,2	8,2	8	9,7
olka	k	2005	2011	cukor-gurgul5	sroczka	8,3	7,5	8	7,3	8,3
orchidea-o	k	2005	2011	ousor VIII	wołga	9	8,5	8	8,2	9
ozi	o	2007	2011	goral	wołga	8,8	8	7,8	7,3	8,3
powiew	o	2000	2011	hroby V	polanka	7,7	7,7	8	7,5	9,5
sawa	k	2000	2011	goral	polanka	8	7,3	7,7	7,3	8,7
suzi	k	2005	2011	pietrosu VI	sroczka	8,2	7,2	8,3	8,2	8,7
szum	w	2007	2011	pietrosu VI	sroczka	8,2	7,8	8,3	7,8	9,3
walencja	k	2007	2011	hroby V	wyderka	9	7,5	7,7	8	9,3
wanessa	k	2007	2011	goral	wyderka	8,5	7,5	7,7	8,2	9
wars	o	2007	2011	goral	wyderka	8,5	7,3	7,7	7	8,3
wataha	k	2007	2011	pietrosu VI	wyderka	8,7	7,7	8,3	8,3	9,5
ważniak	o	2000	2011	hroby V	wyderka	9,2	8	8	8	8,7
wega	k	2002	2011	cukor-gurgul5	sroczka	9,5	8,2	8,5	8,3	9,5
wiraż	o	2005	2011	hroby V	wyderka	9,3	8,5	8,5	8,5	9,5
antena	k	2001	2012	ousor VIII	jagoda	8,1	8	7,7	8	8,6
azalia-w	k	2006	2012	ousor VIII	agatka	8,8	8,2	8,2	8,2	8,8
cisawa	k	2007	2012	pietrosu VI	czeremcha	9,5	8	8,2	8	9,1
fifka	k	2007	2012	hroby V	wrona	8,2	7,5	8,1	7,9	8,3
fiona	k	2008	2012	pietrosu VI	wrona	8,5	8	8	8	8,8

genewa	k	2008	2012	hroby V	gurgul V-23	8	8	7,7	8,1	8,4
głuszec	w	2002	2012	hroby V	goral III-4	7,6	8	7,7	7,5	8,2
grabina	k	2006	2012	hroby V	goral III-4	8,9	8	8,8	8,1	8,8
ifa	k	2007	2012	hroby V	jagoda	8	7,6	8,5	7,8	8,2
istria	k	2007	2012	hroby V	jagoda	8,9	7,8	8,5	7,9	8,9
laguna-o	k	2005	2012	hroby V	laliszka	8,2	7,6	7,9	7,5	8,4
lokaj z bacówki	w	2007	2012	hroby V	laliszka	7,5	7,5	7,3	7,9	8,1
lotka	k	2003	2012	ousor VIII	laliszka	8,9	8,1	8,5	8	8,3
lupo	o	2005	2012	polan	laliszka	8,1	7,5	7,7	7,7	8,4
neron	w	2003	2012	hroby V	nakoneczna	7,9	8	7,1	7,5	8,4
obawa	k	2002	2012	hroby V	wołga	7,5	7,5	7,5	7,6	8,5
ogień	o	2007	2012	hroby V	wołga	8,2	8	7,5	7,1	8,4
okowita	k	2003	2012	cukor-gurgul5	wołga	8	8,1	7,6	7,7	8,4
olgierd-o	o	2005	2012	ousor VIII	wołga	9,2	8	8,3	8,2	9,2
opończa	k	2003	2012	pietrosu VI	wołga	8,5	8	7,9	7,9	8,6
ozi	o	2007	2012	goral	wołga	8,4	7,5	7,4	7,7	8,4
pako	o	2008	2012	prislop	polanka	8,1	7,5	7,9	8	8,7
patka	k	2006	2012	hroby V	polanka	8,6	8,1	7,9	8,1	8,9
pesetka	k	2006	2012	hroby V	polanka	9,5	8,2	8	8	9,3
polatucha	k	2006	2012	goral	polanka	8,4	7,9	7,7	7,9	8,5
potencjał	o	2008	2012	hroby V	polanka	9,2	8,4	7,5	7,3	8,5
selma	k	2006	2012	pietrosu VI	sroczka	8	7,5	8	7,7	8,1
suzi	k	2005	2012	pietrosu VI	sroczka	8,5	7,8	8	8,2	8,6
wieśniak	o	2007	2012	hroby V	wyderka	9,3	8,1	8,1	7,7	9
willa-kur	k	2007	2012	goral	wyderka	8,1	7,6	6,8	6,9	8
wizjer	w	2007	2012	pietrosu VI	wyderka	8,3	8	8,1	8,4	8,7
amfa	k	2005	2013	cukor-gurgul5	agatka	7,6	7	7,4	7,4	7,9
Auna	k	2008	2013	goral	agatka	8,5	8	7,7	8,5	8,8
bahama	k	2007	2013	hroby V	bajkałka	8,4	7,9	7,6	7,6	8,5
Bojko	o	2008	2013	hroby V	bajkałka	8,5	8,4	7,5	7,6	8,5
cisawa	k	2007	2013	pietrosu VI	czeremcha	9,7	8,3	8,1	8	9,3
fabrina	k	2005	2013	goral	wrona	8	7,5	8	7,2	8,3
fala	k	2003	2013	hroby V	wrona	8,4	8	7,8	8	8,4
forteca	k	2008	2013	goral	wrona	9,4	8,4	7,5	8,4	9,2

galera	k	2008	2013	pietrosu VI	gurgul V-23	8,7	7,9	8	7,9	8,9
genewa	k	2008	2013	hroby V	gurgul V-23	8,4	8	7,5	7,9	8,6
grużа	k	2009	2013	hroby V	gurgul V-23	8,5	8	7,5	7,8	8,5
jagła	k	2006	2013	goral	jagoda	9,3	8,3	7,4	8,2	8,9
lando-o	o	2004	2013	hroby V	laliszka	9,4	8,3	8,4	8	9,1
lena	k	2009	2013	hroby V	laliszka	8,5	8	8,1	7,8	8,6
liga	k	2008	2013	ousor VIII	laliszka	8	8,1	8,1	8,2	8,7
litria	k	2004	2013	cukor-gurgul5	laliszka	8,6	8	7,1	7,5	8,5
niwa-sonia	k	2007	2013	pietrosu VI	nakoneczna	8,5	7,8	7,9	8,1	8,5
ogień	o	2007	2013	hroby V	wołga	8,1	7,7	7,9	7,6	8,5
okowita	k	2003	2013	cukor-gurgul5	wołga	9,1	8,4	8,3	7,7	9,1
oliwka	k	2006	2013	polan	wołga	9	7,9	7,5	7,9	8,9
opończa	k	2003	2013	pietrosu VI	wołga	8,9	8,2	7,9	8,3	9
orka-kur	k	2009	2013	hroby V	wołga	8,3	7,6	8	8,1	7,9
otto	o	2000	2013	goral	wołga	8,9	7,9	7,6	8,3	9
owa-kur	k	2007	2013	goral	wołga	7,5	7,9	7,6	7,7	8,4
ozi	o	2007	2013	goral	wołga	8,6	7,7	8	8	8,5
pamirka-tabun	k	2008	2013	pietrosu VI	polanka	8,2	8	7,3	7,6	8,5
patka	k	2006	2013	hroby V	polanka	9,2	8,2	7,9	8,9	9,4
peluszka	k	2004	2013	goral	polanka	8	7,9	8,1	7,8	8,5
pesetka	k	2006	2013	hroby V	polanka	9,1	8,2	7,7	8,3	9
piątek	w	2009	2013	cukor-gurgul5	polanka	8,3	7,9	6,7	7,1	7,7
pola	k	2006	2013	cukor-gurgul5	polanka	7,7	8	7,3	7,6	8,4
polatucha	k	2006	2013	goral	polanka	8,9	8	8,3	8,3	9,1
prehyba	k	1999	2013	cukor-gurgul5	polanka	8,8	8,2	7,4	8,3	8,8
wega	k	2002	2013	cukor-gurgul5	sroczka	9,3	8,2	8,3	7,8	9,1
werona	k	2009	2013	hroby V	wyderka	8,1	8	8	8	8,3
wieśniak	o	2007	2013	hroby V	wyderka	9,1	8	7,5	8,1	8,8
wiśnicz	o	2008	2013	pietrosu VI	wyderka	8,7	7,9	8	8,2	9
wizjer	w	2007	2013	pietrosu VI	wyderka	7,7	8,1	8,4	8,5	8,5
wrzos-w	o	2009	2013	pietrosu VI	wyderka	9,1	8	8,4	7,8	9
alex	o	2009	2014	hroby V	agatka	8,3	7,8	7,4	7,9	8,8
antaba	k	2004	2014	cukor-gurgul5	agatka	9	8,4	7,5	7,6	9
azalia-w	k	2006	2014	ousor VIII	agatka	9	8	8,4	8,4	9

cisawa	k	2007	2014	pietrosu VI	czeremcha	9,5	8,1	7,7	7,7	9,2
czarka	k	2009	2014	pietrosu VI	czeremcha	8	7,7	7,5	7,5	8,5
farunka	k	2007	2014	polan	wrona	8,1	7,7	7,4	7,7	8,4
forteca	k	2008	2014	goral	wrona	9	8,3	7,6	8	8,9
furora	k	2009	2014	pietrosu VI	wrona	9,1	7,9	8,4	8,4	9,2
genewa	k	2008	2014	hroby V	gurgul V-23	8,3	8,3	8,5	8	9,1
gniewczyna	k	2008	2014	hroby V	gurgul V-23	7,7	7,5	7,6	7,4	8
grabina	k	2006	2014	hroby V	goral III-4	8,3	7,9	8,2	7,9	9
ifa	k	2007	2014	hroby V	jagoda	8	7,7	8,4	7,7	8,5
istria	k	2007	2014	hroby V	jagoda	9,1	8,4	8,2	7,8	9,2
jagła	k	2006	2014	goral	jagoda	9	8,5	8,4	8	9,2
lando-o	o	2004	2014	hroby V	laliszka	9,4	8,3	7,8	8,5	9,4
lena	k	2009	2014	hroby V	laliszka	9,2	8,4	8,4	8,2	9,3
lotka	k	2003	2014	ousor VIII	laliszka	8,9	8	8,8	8,6	8,9
niwa-sonia	k	2007	2014	pietrosu VI	nakoneczna	8,5	7,8	7,6	7,7	8,5
obca-w	k	2010	2014	goral	wołga	8	7,4	7,9	7,8	8,4
ogień	o	2007	2014	hroby V	wołga	8,3	8	7,7	7,6	8,7
okowita	k	2003	2014	cukor-gurgul5	wołga	9,1	8,5	7,8	8,3	9,2
olcha-w	k	2003	2014	pietrosu VI	wołga	7,9	7,8	7,4	7,6	8,5
opończa	k	2003	2014	pietrosu VI	wołga	8,5	8	7,7	7,9	8,8
otto	o	2000	2014	goral	wołga	8,4	7,9	7,4	7,6	8,7
ozi	o	2007	2014	goral	wołga	8,3	7,9	7,5	8	8,8
padżerka	k	2008	2014	pietrosu VI	polanka	8,6	8,1	7,3	7,7	8,8
pamirka-tabun	k	2008	2014	pietrosu VI	polanka	8,5	7,9	7,3	7,3	8,7
piątek	w	2009	2014	cukor-gurgul5	polanka	8	7,8	7,6	7,3	8,3
polatucha	k	2006	2014	goral	polanka	8,5	8	7,6	8	8,8
Poter	o	2003	2014	ousor VIII	polanka	8,9	7,9	8,3	7,9	9,1
ptysia	k	2009	2014	pietrosu VI	polanka	8	7,9	7,2	8,4	8,1
selma	k	2006	2014	pietrosu VI	sroczka	8,1	7,7	8,1	7,8	8,6
wicehrabina	k	2008	2014	cukor-gurgul5	wyderka	8,3	8	7,6	7,7	8,6
widok	o	2009	2014	hroby V	wyderka	8,4	8,5	7,8	8	9,1
wika	k	2008	2014	hroby V	wyderka	8	7,6	6,7	7,3	8,2
wiśnicz	o	2008	2014	pietrosu VI	wyderka	8,2	8	8,4	8,2	9
wrzos-w	o	2009	2014	pietrosu VI	wyderka	9,2	8,1	8,4	8	9,2

amant-w	o	2008	2015	hroby V	agatka	8	7,5	7,3	7,3	8,5
Auna	k	2008	2015	goral	agatka	8,9	8	7,7	7,6	8,9
azalia-w	k	2006	2015	ousor VIII	agatka	8,7	8	8,1	8,4	8,9
Bojko	o	2008	2015	hroby V	bajkałka	8,7	8	7,9	8,2	8,9
cyfra	k	2009	2015	goral	czeremcha	8,9	8	8,1	7,8	8,8
czarka	k	2009	2015	pietrosu VI	czeremcha	8,4	8	7,3	7,5	8,6
czerszla	k	2008	2015	goral	czeremcha	9	8,1	7,7	7,9	9,1
jasyr	o	2011	2015	hroby V	jagoda	7,9	7,8	8	8,1	8,5
jesion	o	2011	2015	pietrosu VI	jagoda	7,8	7,3	7,3	7,5	8,1
Judym	o	2010	2015	polan	jagoda	8,9	8	8,8	8,3	9,6
lawenda	k	2004	2015	cukor-gurgul5	wrona	8,5	8	7,1	7,5	8,3
lennik	w	2005	2015	hroby V	laliszka	8,4	7,9	7,2	7,6	8,9
liryka	k	2009	2015	cukor-gurgul5	laliszka	8	7,7	7,5	8	8,4
lotek	o	2011	2015	polan	laliszka	8,4	7,5	8,4	7,8	8,2
lotka	k	2003	2015	ousor VIII	laliszka	8,5	7,8	7,9	8	8,7
odessa-w	k	2010	2015	goral	wołga	7,6	7,1	6,8	7,5	7,7
odwaga-kur	k	2011	2015	goral	wołga	8,3	8,1	8	7,4	8,5
oficer	o	2005	2015	hroby V	wołga	8	7,6	8	7,5	8,2
ogień	o	2007	2015	hroby V	wołga	8,5	8	7,6	7,9	8,5
otto	o	2000	2015	goral	wołga	9,1	8	7,6	7,5	8,8
ozi	o	2007	2015	goral	wołga	8,5	7,9	7,5	7,4	8,6
pako	o	2008	2015	prislop	polanka	8,9	7,7	7,6	7,4	8,7
pamirka-tabun	k	2008	2015	pietrosu VI	polanka	9	8	7,9	7,7	9
paulinka-o	k	2007	2015	goral	polanka	9	8,1	7,7	7,7	8,6
piątek	w	2009	2015	cukor-gurgul5	polanka	7	8	8	7,6	8,6
polatucha	k	2006	2015	goral	polanka	8,9	8	8	7,7	8,8
Poter	o	2003	2015	ousor VIII	polanka	9,3	8	8,3	7,9	9,1
praga gj	k	2010	2015	cukor-gurgul5	polanka	7,9	7,4	6,8	7,4	8,1
prehyba II	k	2010	2015	ousor VIII	polanka	7	7,2	7,2	7,7	8
ptysia	k	2009	2015	pietrosu VI	polanka	7,7	7,4	6,8	7,7	7,7
selma	k	2006	2015	pietrosu VI	sroczka	8,1	7,8	8	8,3	8,8
skawa	k	2010	2015	hroby V	sroczka	8,7	8,3	7,9	8,3	9,2
suzi	k	2005	2015	pietrosu VI	sroczka	9	8,1	8,1	8,5	8,9
wieśniak	o	2007	2015	hroby V	wyderka	9,2	8	7,5	7,7	8,9

wika	k	2008	2015	hroby V	wyderka	8	7,6	7	7,4	8,4
z-olisa	k	2005	2015	pietrosu VI	reda	8,5	8	7,5	7,5	8,7
azalia-w	k	2006	2016	ousor VIII	agatka	9,2	8,5	8,7	7,9	9,4
azja-w	o	2011	2016	cukor-gurgul5	agatka	8,3	8	7,6	7,2	8,3
buzia	k	2009	2016	ousor VIII	bajkałka	8,5	8	7,9	7,7	8,7
czerszla	k	2008	2016	goral	czeremcha	8,4	8	7,7	8	8,6
forteca	k	2008	2016	goral	wrona	9,2	8,2	7,5	8,3	9
furora	k	2009	2016	pietrosu VI	wrona	9,5	8,6	8	8,6	9
galera	k	2008	2016	pietrosu VI	gurgul V-23	9,2	8,2	7,9	8,4	9,3
gniewczyna	k	2008	2016	hroby V	gurgul V-23	8,5	8	7,9	7,8	8,9
grabina	k	2006	2016	hroby V	goral III-4	8,5	8,1	8,4	8,1	9
jasyr	o	2011	2016	hroby V	jagoda	8,9	8	8,2	8,2	9
Judym	o	2010	2016	polan	jagoda	9	8,5	8,6	8,6	9,5
kupidyn z sadyby	o	2008	2016	polan	cocha	8,5	7,6	7,3	7,7	8,4
lando-o	w	2004	2016	hroby V	laliszka	8,5	8,3	7,9	8,1	9
leksja-o	k	2004	2016	hroby V	laliszka	8,9	8	8	8,5	9,5
lena	k	2004	2016	cukor-gurgul5	laliszka	8,1	7,5	7,8	8	8,5
liryka	k	2009	2016	cukor-gurgul5	laliszka	8,3	7,7	7,9	7,9	8,9
lotek	o	2011	2016	polan	laliszka	8,3	7,9	8,1	8,1	8,4
lotka	k	2003	2016	ousor VIII	laliszka	8,9	8,1	8,2	8	9
lotka	k	2003	2016	ousor VIII	laliszka	8,7	8,4	7,8	7,9	9,1
neron	w	2003	2016	hroby V	nakoneczna	7,9	7,6	7,6	7,7	8,6
nicea	k	2010	2016	goral	nakoneczna	8	7,6	7,1	7,5	8,1
oaza-o	k	2011	2016	goral	wołga	8,3	7,9	7,8	7,9	8,5
obelix-kur	w	2011	2016	hroby V	wołga	8,1	8,1	8,4	8,3	8,9
odwaga-kur	k	2011	2016	goral	wołga	8,8	8,3	8,1	7,7	8,9
oficer	o	2005	2016	hroby V	wołga	8,3	7,9	7,4	7,7	9
ogień	o	2007	2016	hroby V	wołga	8,9	8,5	7,3	7,5	8,7
okowita	k	2003	2016	cukor-gurgul5	wołga	9,2	8,4	7,9	8,3	9,2
opończa	k	2003	2016	pietrosu VI	wołga	8,8	8,3	8,2	7,9	9,2
ozi	o	2007	2016	goral	wołga	8,9	8,3	7,9	7,8	9,1
patka	k	2006	2016	hroby V	polanka	9,1	8,5	8	8,4	9,4
pędziwiatr	o	2007	2016	prislop	polanka	8,4	7,6	8,5	8,3	8,9
piątek	w	2009	2016	cukor-gurgul5	polanka	8	8	7,8	7,7	8,7

proza-o	k	2009	2016	ousor VIII	polanka	8,5	8	7,5	7,7	8,9
puszta z brzezia	k	2009	2016	pietrosu VI	polanka	8,6	8	7,7	7,5	8,8
skawa	k	2010	2016	hroby V	sroczka	9	8,2	7,7	8,1	8,9
suzi	k	2005	2016	pietrosu VI	sroczka	8,3	7,8	7,8	8	8,6
wag-w	o	2012	2016	goral	wyderka	9	8,3	7,5	7,3	8,6
werona	k	2009	2016	hroby V	wyderka	8,9	8,4	8	7,7	8,7
wieróżka	k	2012	2016	ousor VIII	wyderka	9	8,3	8	7,9	9
wrzos-w	o	2009	2016	pietrosu VI	wyderka	8,6	8,5	8,4	8	9,4
abra gj	k	2012	2017	cukor-gurgul5	agatka	8	7,9	7,4	7,3	8,2
alpejczyk	w	2009	2017	pietrosu VI	agatka	8,1	7,9	7,4	7,2	8,7
arktyka-w	k	2012	2017	goral	agatka	8,1	7,9	8,3	8,5	9,3
bajadera	k	2013	2017	hroby V	bajkałka	8,5	8,4	7,7	8	8,7
cyrkonia	k	2011	2017	pietrosu VI	czeremcha	7,7	7,5	7,9	7,7	8,5
czarka	k	2009	2017	pietrosu VI	czeremcha	8,2	7,8	7,9	7,9	8,7
czerszla	k	2008	2017	goral	czeremcha	8,5	8	7,4	7,8	8,6
istria	k	2007	2017	hroby V	jagoda	9,2	7,9	7,4	7,5	8,7
Judym	o	2010	2017	polan	jagoda	9,1	8,4	8,8	9,3	9,7
lando-o	w	2004	2017	hroby V	laliszka	8,6	8,2	7,2	7,1	8,8
lennik	w	2005	2017	hroby V	laliszka	8,5	8,3	7,6	7,5	8,9
lotka I	k	2003	2017	goral	laliszka	9,2	8,3	7,8	8	9,2
loża	k	2012	2017	pietrosu VI	laliszka	8,8	7,9	7,9	7,9	9,1
nasir	o	2003	2017	cukor-gurgul5	nakoneczna	9,6	8,4	7,8	8,2	9,7
neron	w	2003	2017	hroby V	nakoneczna	8	8	7,6	7,5	8,5
obelix-kur	w	2011	2017	hroby V	wołga	8,1	7,9	7,9	8,4	8,9
odwaga-kur	k	2011	2017	goral	wołga	8,8	7,9	7,8	7,8	8,8
oficer	o	2005	2017	hroby V	wołga	7,9	7,5	7,4	7,6	8,8
ogień	o	2007	2017	hroby V	wołga	9	8,2	7,7	7,7	9
okowita	k	2003	2017	cukor-gurgul5	wołga	9,3	8,2	8,1	8,1	9,3
opończa	k	2003	2017	pietrosu VI	wołga	8,7	8	8	8,1	9,1
orawa	k	2009	2017	hroby V	wołga	9	8	7,4	7,9	9
orka-kur	k	2009	2017	hroby V	wołga	8,5	8	7,9	7,4	9
ozi	o	2007	2017	goral	wołga	8,7	8	7,6	7,5	8,9
pączek	o	2012	2017	goral	polanka	8,3	8	6,5	6,9	8,5
peira	k	2012	2017	hroby V	polanka	9,3	8,3	7,9	8,5	9,4

pędziwiatr	o	2007	2017	prislop	polanka	8,5	7,6	8,5	8,3	8,5
pretoria	k	2007	2017	hroby V	polanka	8,4	7,8	7,9	8,1	8,3
ptysia	k	2009	2017	pietrosu VI	polanka	7,8	7,4	7,5	7,8	8,5
selma	k	2006	2017	pietrosu VI	sroczka	8,6	8	7,8	8	8,7
suzi	k	2005	2017	pietrosu VI	sroczka	8,5	8	7,4	7,8	8,8
wella	k	2010	2017	hroby V	wyderka	8,4	7,8	7,4	7,5	8,7
werona	k	2009	2017	hroby V	wyderka	9	8,3	7,6	8	8,8
wichren	w	2008	2017	goral	wyderka	8	7,9	7,8	7,6	8,3
wieróżka	k	2012	2017	ousor VIII	wyderka	8,9	8	7,7	8,1	9
wisky	k	2009	2017	hroby V	wyderka	8,6	8	7,6	7,4	8,6
wróżbitka	k	2011	2017	hroby V	wyderka	8,8	8,2	7,4	7,7	9
alpejczyk	w	2009	2018	pietrosu VI	agatka	8	7,9	7,1	7,6	8,5
ankara-w	k	2014	2018	goral	agatka	8,5	8,1	7,9	8,4	9
arizona	k	2009	2018	pietrosu VI	agatka	9	7,9	8,4	7,9	9,3
azalia-w	k	2006	2018	ousor VIII	agatka	9,3	8,3	8,5	8,5	9,5
czapla	k	2008	2018	goral	czeremcha	8	7,7	7,5	8	8,6
gerta	k	2010	2018	ousor VIII	ousor	8,6	8	7,9	7,8	9
grabina	k	2006	2018	hroby V	goral III-4	8,3	7,8	8,3	8,2	9
jedlina	k	2012	2018	hroby V	jagoda	8	7,5	7,5	7,9	8,4
jeżyk	o	2013	2018	polan	jagoda	8	7,5	7,6	8	9
Judym	o	2010	2018	polan	jagoda	9,1	8	8,6	8,9	9,6
leksja-o	k	2004	2018	hroby V	laliszka	9,1	8	8	8,1	9,3
lennik	w	2005	2018	hroby V	laliszka	8,4	8,2	7,3	7,8	8,7
lokaj z bacówki	w	2007	2018	hroby V	laliszka	8	8	7,4	7,5	8,6
lotek	o	2011	2018	polan	laliszka	7,3	7,2	8	7,1	7,7
loża	k	2012	2018	pietrosu VI	laliszka	9,1	8,1	7,7	7,9	9,3
oaza-o	k	2011	2018	goral	wołga	8,5	8	7,8	8,4	9
ogień	o	2007	2018	hroby V	wołga	8,8	8,1	7,4	7,5	8,7
opończa	k	2003	2018	pietrosu VI	wołga	8,9	8,4	7,9	8	9
orawa	k	2009	2018	hroby V	wołga	8,5	8	7,5	8	8,7
orka-kur	k	2009	2018	hroby V	wołga	8,5	8	7,7	7,9	8,9
ozi	o	2007	2018	goral	wołga	8,5	8	7,9	7,5	8,9
ozyr	w	2008	2018	goral	wołga	8,5	8,1	7,7	7,8	9
pączek	o	2012	2018	goral	polanka	7,9	7,6	7,4	7,4	8,6

pędziwiatr	o	2007	2018	prislop	polanka	8,5	7,7	8,3	8	8,9
pokuska	k	2012	2018	goral	polanka	8,5	7,8	7,9	7,5	8,7
puszta z brzezia	k	2009	2018	pietrosu VI	polanka	9,1	8,2	8	7,5	9,2
serbia	k	2009	2018	pietrosu VI	sroczka	8,5	8	7,7	8	9
skawa	k	2010	2018	hroby V	sroczka	8,7	8	7,9	8,1	9,2
syrenka	k	2013	2018	hroby V	nakoneczna	8,5	8	8	8	9
Świteź	w	2011	2018	goral	wyderka	8	8	7	7,5	8,6
wieróżka	k	2012	2018	ousor VIII	wyderka	9,2	7,7	7,9	8	9,4
arizona	k	2009	2019	pietrosu VI	agatka	9,4	8,4	8,4	7,9	9,3
azalia-w	k	2006	2019	ousor VIII	agatka	9,5	8,4	8,2	8,1	9,6
bols	o	2013	2019	hroby V	sroczka	8,6	7,8	7,1	7,5	8,4
brodka z izb	k	2013	2019	hroby V	bajkałka	9	8	7,9	7,9	9,2
ceregiela	k	2014	2019	pietrosu VI	czeremcha	8,7	8,1	7,7	7,8	9,1
czapla	k	2008	2019	goral	czeremcha	8,5	7,8	7,6	8,3	8,9
czarka	k	2009	2019	pietrosu VI	czeremcha	8,5	7,7	7,5	7,8	9
grom	o	2007	2019	hroby V	pietrosu	8,6	8	7,7	7,6	8,9
jedlina	k	2012	2019	hroby V	jagoda	8,2	7,6	7,5	6,9	8
jeżyk	o	2013	2019	polan	jagoda	8,4	8	7,7	8,4	9,2
jubiler	o	2014	2019	polan	jagoda	9	8,4	8	7,9	9,2
jugla	k	2013	2019	pietrosu VI	jagoda	9,1	8	7,9	8,2	9,1
liga	k	2008	2019	ousor VIII	laliszka	8,6	8	7,7	8	9
loża	k	2012	2019	pietrosu VI	laliszka	9,2	8,4	7,8	8,3	9,4
obca-w	k	2010	2019	goral	wołga	8	7,8	7,4	7,3	8,3
ogień	o	2007	2019	hroby V	wołga	9	8	7,9	7,7	9,1
orawa	k	2009	2019	hroby V	wołga	8,3	7,9	7,1	7,4	8,4
oregona	k	2012	2019	hroby V	wołga	8,3	7,6	7,2	7,8	8,4
pączek	o	2012	2019	goral	polanka	8	7,6	7,7	7,6	8,4
pędziwiatr	o	2007	2019	prislop	polanka	9,2	8,1	8,3	8,3	9,5
piątek	w	2009	2019	cukor-gurgul5	polanka	8	7,7	7,3	7,5	8,3
pokuska	k	2012	2019	goral	polanka	8,9	8,1	8	7,9	9
pretoria	k	2007	2019	hroby V	polanka	8,2	7,5	7,5	7,5	8,5
suzi	k	2005	2019	pietrosu VI	sroczka	8,3	7,6	7,5	7,8	8,6
Świteź	w	2011	2019	goral	wyderka	8	7,8	7,4	7,7	8,4
wieróżka	k	2012	2019	ousor VIII	wyderka	9,1	8	7,8	8,1	9,4

wiki	k	2008	2019	cukor-gurgul5	wyderka	8,6	7,5	7,8	8	8,9

total	Hucul path	endur.-con	total	place
37	73,5	0	110,5	19
40,6	11	49,62	101,22	24
41,6	66,5	48,77	156,87	6
38,4	0	38,45	76,85	27
38,2	29	36,55	103,75	22
45	0	0	45	30
38,2	80	34,26	152,46	10
38,6	54,5	55,8	148,9	11
40,4	49	36,77	126,17	16
41,2	0	62,43	103,63	23
42,4	58	0	100,4	25
39,6	74	40,34	153,94	7
43,8	0	0	43,8	31
39	51,5	49,47	139,97	13
38,4	68	28,29	134,69	14
43,2	67	0	110,2	20
39,2	0	37,05	76,25	28
41,8	74	47,88	163,68	3
38,4	0	44,83	83,23	26
35,6	0	0	35,6	33
38,2	64,75	28,29	131,24	15
34,6	0	0	34,6	34
41,4	68	61,64	171,04	2
36,6	39,5	49,62	125,72	17
43,2	80	48,07	171,27	1
45,4	67,75	47,88	161,03	4
38,8	64,5	50,46	153,76	8
41,2	32	34,91	108,11	21
41,8	73	27,4	142,2	12
42,8	74	36,55	153,35	9
40,6	34,5	35,86	110,96	18
41,8	62	55,12	158,92	5
40,6	0	0	40,6	32

37,6	0	27,93	65,53	29
40	64,5	31,79	136,29	23
37	0	21,82	58,82	34
37,17	61,5	46,68	145,35	20
41,17	50,25	29,71	121,13	26
38,67	0	17,82	56,49	35
40,5	38,5	41,11	120,11	27
40,67	35	41,15	116,82	29
39,67	42	19,38	101,05	31
39,17	0,5	40,4	80,07	33
39,33	70	50,01	159,34	6
41,33	72	56,47	169,8	4
40,17	57,5	48,94	146,61	19
44,2	50,75	45,13	140,21	21
38,17	67,5	50,8	156,47	9
43,83	65	29,77	138,6	22
39,17	74	65,43	178,6	1
38,83	80	57,73	176,46	2
43,33	64	21,26	128,59	24
41,67	80	52,65	174,32	3
43,5	65,25	53,87	162,62	5
37,83	74	45,25	157,08	7
40,2	73,5	41,21	155,04	13
41,17	53,75	32,3	127,22	25
38,83	74	36,28	147,11	17
41,33	57,25	0	98,58	32
44	72,25	33,08	149,33	16
39	70,75	46,03	155,78	10
39,5	67	48,94	155,44	11
42	73	41,77	156,77	8
41,17	64	49,89	155,06	12
33,5	36,5	48,9	118,9	28
40,67	63,5	45,64	149,81	14
40,83	64,75	43,21	148,79	18

44,17	76	33,64	153,8	6
40,33	36,75	28,53	105,61	22
41,33	72	58,23	171,56	1
41	14	0	55	27
40,5	70	29,77	140,27	10
42,5	77	20,21	139,71	11
40,83	80	0	120,83	16
39,67	80	40,34	160	4
41,5	79	32,84	153,34	7
39,5	68	0	107,5	20
42,17	80	0	122,17	15
37,83	61,5	39,26	138,59	12
38,67	80	50,79	169,46	2
42	62,5	0	104,5	24
43,17	77,5	0	120,67	17
39,5	0	39,71	79,21	25
42,67	80	27,31	149,98	8
40,33	72	47,09	159,42	5
40,33				28
39	73,75	23,13	135,88	14
40,5	56,5	39,69	136,69	13
41,5	63,5	0	105	23
41,5	76	24,62	142,12	9
40,83	75,5	0	116,33	18
38,83	0	33,12	71,95	26
42,5	50	22,31	114,81	19
41,83	65	0	106,83	21
44	76	46,58	166,58	3
44,33				28
40,4	66,5	53,33	160,23	8
42,2	70,25	62,22	174,67	5
42,8	27,5	17,78	88,08	24
40	39,75	0	79,75	25
41,3	44,25	53,33	138,88	20

40,2	57	44,44	141,64	16
39	28,25	71,11	138,36	22
42,6	69	80	191,6	3
40,1	0	17,78	57,88	30
42	65,75	53,33	161,08	7
39,6	68	0	107,6	23
38,3	74	26,67	138,97	12
41,7	72,5	17,78	131,98	17
39,4	0	0	39,4	31
38,9	65,5	44,44	148,84	10
38,6	0	35,56	74,16	29
39,2	47,5	71,11	157,81	11
39,8	66,25	35,56	141,61	14
42,9	0	53,33	96,23	26
40,9	72,25	62,22	175,37	4
39,4	59,75	53,33	152,48	9
40,2	52,5	53,33	146,03	15
41,6	59	35,56	136,16	19
43	67,5	62,22	172,72	6
40,4	80	71,11	191,51	1
40,9	25,5	0	66,4	27
39,3	67,25	35,56	142,11	13
41,1	0	35,56	76,66	28
42,2	77	71,11	190,31	2
37,4	63	26,67	127,07	21
41,5	71	17,78	130,28	18
37,3	0	44,44	81,74	31
41,5	25,25	35,56	102,31	23
40	41,5	0	81,5	25
40,5	39,75	17,78	98,03	21
43,4	0	44,44	87,84	30
39	42,75	35,56	117,31	19
40,6	58,75	53,33	152,68	12
42,9	72	44,44	159,34	9

41,4	80	44,44	165,84	4
40,4	52	53,33	145,73	16
40,3	44,5	44,44	129,24	18
42,1	0	53,33	95,43	28
43,2	0	17,78	60,98	32
41	0	62,22	103,22	26
41,1	0	0	41,1	37
39,7	49	17,78	106,48	20
40,8	5,74	0	46,54	34
39,8	77,25	53,33	170,38	2
42,6	80	44,44	167,04	3
41,2	58,25	44,44	143,89	15
42,3	80	53,33	175,63	1
39,9	69,25	35,56	144,71	14
41,7	80	0	121,7	17
39,1	35	17,78	91,88	24
40,8	49	0	89,8	22
39,6	0	62,22	101,82	27
43,6	0	44,44	88,04	29
40,3	0	0	40,3	38
42,3	58	53,33	153,63	10
37,7	0	17,78	55,48	33
39	0	0	39	39
42,6	57,5	53,33	153,43	11
41,5	0	0	41,5	36
42,7	63,75	62,22	168,67	6
40,4	72	53,33	165,73	5
41,5	58	53,33	152,83	13
41,8	69	53,33	164,13	7
41,2	69	53,33	163,53	8
42,3	0	0	42,3	35
40,2	40,75	80	160,95	26
41,5	45,5	80	167	23
42,8	77	80	199,8	2

42,2	32	71,11	145,31	32
39,2	27	80	146,2	33
39,3	0	62,22	101,52	37
41,8	80	62,22	184,02	8
43	60	80	183	15
42,2	60	80	182,2	16
38,2	73	80	191,2	6
41,3	71	80	192,3	7
40,3	0	80	120,3	34
42,7	68	80	190,7	9
43,1	66	80	189,1	10
43,4	72	62,22	177,62	13
43,5	58,5	80	182	17
43,2	37	80	160,2	28
40,1	65	80	185,1	14
39,5	53,5	80	173	19
40,3	72,5	71,11	183,91	11
42,9	80	80	202,9	1
39,2	41	80	160,2	27
40,9	72	80	192,9	4
40	72	71,11	183,11	12
40,5	30	80	150,5	30
40,5	45,25	0	85,75	35
39,7	31,25	80	150,95	29
39	73	80	192	5
40,9	78	80	198,9	3
42,1	50	80	172,1	21
39,6	52	80	171,6	20
40,3	29	80	149,3	31
40,2	58,25	80	178,45	18
41,8	41,5	80	163,3	25
37,8	58,25	62,22	158,27	24
41,8	52,5	71,11	165,41	22
42,9	0	62,22	105,12	36

38,6	46	62,22	146,82	25
41,1	72	80	193,1	1
41,1	0	71,11	112,21	33
41,7	63	80	184,7	5
41,6	57	0	98,6	28
39,8	38,25	80	158,05	22
41,8	54,5	62,22	158,52	19
40,3	69	71,11	180,41	6
38	62,5	80	180,5	8
43,6	33,5	80	157,1	23
39,4	65	62,22	166,62	15
40	56,5	62,22	158,72	18
39,6	45	80	164,6	20
40,3	0	62,22	102,52	36
40,9	49,75	80	170,65	17
36,7	37,5	80	154,2	24
40,3	0	71,11	111,41	34
39,3	70,5	62,22	172,02	10
40,5	57	80	177,5	13
41	70,75	80	191,75	2
39,9	65	80	184,9	4
40,3	0	80	120,3	21
41,6	28	80	149,6	26
41,1	52,25	0	93,35	29
40,2	22,5	62,22	124,92	27
41,4	62	62,22	165,62	16
42,6	20	44,44	107,04	30
37,6	0	80	117,6	32
37,1	61,5	80	178,6	9
37,3	57,5	80	174,8	14
41	39	0	80	35
42,4	61	44,44	147,84	21
42,6	67	80	189,6	3
41,3	58,5	80	179,8	11

38,4	64	80	182,4	7
40,2	59	80	179,2	12
43,7	0	44,44	68,85	35
39,4	0	44,44	64,85	36
40,8	0	80	92,64	29
40,7	18,5	62,22	102,35	27
42,2	52,5	62,22	146,05	12
43,7	57,5	80	166,84	4
43	71	35,56	149,82	11
41,1	58	71,11	158,71	8
42,1	67	80	177,43	2
42,3	67	17,78	130,92	18
44,2	69,5	8,89	128,9	20
39,5	34,25	0	74,41	33
41,8	0	17,78	46,77	38
42,9	77	80	190,57	1
39,9	59	80	161,92	6
40,7	35,5	71,11	130,27	19
40,8	49	0	93,89	28
42,2	31,5	80	133,14	17
41,9	28	80	128,52	27
39,4	61	0	107,77	26
38,3	0	62,22	77,31	32
40,4	67	26,67	136,07	15
41,8	59	71,11	160,52	7
41,8	47,25	71,11	145,84	13
40,3	68,75	44,44	151,51	10
40,9	47	71,11	144,8	14
43	68	71,11	172,73	3
42,4	63,5	71,11	166,63	5
42	69	80	179,85	1
43,2	58,75	0	108	25
41,7	52	71,11	151,69	9
40,2	48	0	92,16	30

40,6	37	17,78	92,06	31
40,6	34	80	134,98	16
41,9	65	0	114,77	24
40,5	27,5	80	126,78	21
40,7	27	71,11	138,81	22
41,7	1,5	44,44	68,57	34
42,2	0	26,67	53,76	37
42,9	34	53,33	116,82	23
38,8	39,75	76	137,73	17
39,3	36	80	136,44	18
42,1	48,25	0	93,99	28
41,3	0	76	90,04	30
39,3	7,5	0	40,82	38
40,5	0	76	89,4	32
40,3	0	80	92,24	29
40,7	41,5	72	138,44	16
45,3	61	80	172,49	7
39,9	0	76	88,92	33
40,8	65	0	113,89	24
42,5	61	76	167,25	10
41,6	70	80	180,78	3
43,7	62	0	112,46	25
39,6	57	76	159,93	12
41,2	55	76	158,71	14
41,1	0	76	89,88	31
39,2	68,25	76	173,67	5
41,6	66,25	76	173,09	6
43	64,5	80	175,03	4
41,9	61	80	169,77	9
41,3	63	80	171,79	8
40,8	56	0	102,64	26
40,7	73	76	180,81	2
38,2	0	76	87,56	36
43,4	45	76	147,97	15

41,4	56	76	160,12	11
40,5	28,25	76	124,71	19
39	0	76	88,2	34
41,1	44	0	87,88	35
40,5	25,75	76	121,59	21
39,8	50,5	0	94,97	27
41,7	0	68	84,36	37
39,6	47,5	40	121,06	22
41,7	53	80	159,61	13
40,2	23,25	76	118,22	23
41,1	25,5	76	121,76	20
39,1	23,5	76	117,66	29
41,9	61	80	169,77	2
42,5	37	72	134,25	22
44,1	47,5	60	139,66	20
39,8	39,5	68	132,22	23
41,3	55	80	161,79	5
41,6	33,5	76	132,16	24
39,3	32,5	80	132,07	25
40,1	47	72	144,83	17
44,2	51,5	68	150,74	13
42,5	54	80	161,5	6
40,4	59	64	154,07	11
39,5	55	80	161,35	7
37,3	56,5	72	154,47	10
42,1	47,5	76	150,06	14
41,7	51,5	80	157,74	8
40,5	56	80	162,4	4
42,2	55	48	138,51	21
40,7	14	80	110,06	31
41	44	80	147,8	16
40,8	67	76	173,39	1
41,1	53	72	153,13	12
38,9	30,75	80	129,56	26

41,4	38,75	80	141,56	19
40,4	48	76	149,32	15
42	16	80	113,6	30
41,2	51,75	0	97,65	32
41,9	49	64	142,77	18
41,4	27	80	126,87	28
39,1	61	80	167,53	3
42,2	63	60	157,51	9
43,6	59	68	159,63	14
43,8	54	76	159,54	15
39,4	36,25	64	124,83	23
42	56,25	76	160,91	13
41,4	45	36	116,37	24
41,1	63	76	168,63	8
40,5	54,25	76	157,21	16
40,8	36	68	128,64	22
38,2	20	80	115,56	25
41,7	67	36	144,11	21
42,5	59	76	164,75	10
42,3	54	64	149,34	19
41,3	67	80	176,79	3
43,1	71	36	150,23	17
38,8	41,5	36	109,92	26
41,7	72	80	183,36	2
39,1	62	76	165,78	9
39,3	56	64	149,44	18
39,3	74	80	183,94	1
43,4	63	80	173,47	5
38,8	70	68	169,54	6
41,9	58,75	76	163,96	11
39,2	43,5	80	145,74	20
39,8	29	28	89,09	30
39,3	58	80	163,94	12
42,4	67	76	174,67	4

40,8	61,25	80	169,2	7
