# Supplementary material for: Assessing the utility value of Hucul horses using classification models, based on artificial neural networks
Source: PLoS One. 2022 Jul 26;17(7):e0271340. doi: 10.1371/journal.pone.0271340 (PMC9321442; doi:10.1371/journal.pone.0271340)
Supplement: S1 File — (PDF) [file pone.0271340.s002.pdf]

Information about the network’s architecture

Table 1. Configuration of the tested ANN for predicting horses’ position in competitions

No.	Input layer size	Number of neurons in the 1st hidden layer	Transition function	Number of neurons in the 2nd hidden layer	Transition function	Number of neurons in output layer	Transition function
1	8	8	Tansig	-	-	1	Purelin
2	8	10	Tansig	-	-	1	Purelin
3	8	12	Tansig	-	-	1	Purelin
4	8	7	Tansig	5	Tansig	1	Purelin
5	8	8	Tansig	4	Tansig	1	Purelin
6	8	9	Tansig	3	Tansig	1	Purelin

Table 2. Configuration of the tested ANN for horses’ classification into groups "strong", "medium", "weak"

No.	Input layer size	Number of neurons in the 1st hidden layer	Transition function	Number of neurons in the 2nd hidden layer	Transition function	Number of neurons in output layer	Transition function
1	8	8	Tansig	-	-	3	Purelin
2	8	12	Tansig	-	-	3	Purelin
3	8	16	Tansig	-	-	3	Purelin
4	8	10	Tansig	4	Tansig	3	Purelin
5	8	12	Tansig	6	Tansig	3	Purelin
6	8	12	Tansig	8	Tansig	3	Purelin
7	8	14	Tansig	6	Tansig	3	Purelin
8	8	14	tansig	7	Tansig	3	Purelin
